# Supplementary material for: The evolution of short- and long-range weapons for bacterial competition
Source: Nat Ecol Evol. 2023 Nov 30;7(12):2080–91. doi: 10.1038/s41559-023-02234-2 (PMC10697841; doi:10.1038/s41559-023-02234-2)
Supplement: Supplementary file 1 — Supplementary Tables 1–4, captions for Supplementary Videos and References. [file 41559_2023_2234_MOESM1_ESM.pdf]

nature ecology & evolution

Article https://doi.org/10.1038/s41559-023-02234-2

The evolution of short- and long-range weapons for bacterial competition

In the format provided by the authors and unedited

Supplementary tables

Supplementary Table 1: Model Variables

Variable	Symbol	Units
Cartesian coordinates	x, y	µm
Cell volume fraction field	$\phi(x,y)$	-
Toxin concentration field	$u_T(x,y)$	$kg_T m^{-3}$
For each cell *i*:		
Position vector	$\boldsymbol{p_i} = (p_x, p_y, p_z)_i$	µm
Orientation unit vector	$\boldsymbol{a_i} = (a_x, a_y, a_z)_i$	-
Segment length	L_i	µm
Volume	$V_i = 4\pi R^3/3 + \pi L_i R^2$	$µm^3$
Specific growth rate	$k_{max}(1-ck_{sec})$	h^{-1}

Supplementary Table 2: Model Parameters

Type	Parameter	Symbol / equation(s)	Value(s) [units]	Source
* No specific value assumed; parameter varied implicitly via cluster $\mathbb{D}_T$. ‡ Controlled by N_{hits}.				
Cells	Cell radius	$R = l/2$	0.5 [μm]	[1]
	Cell volume at birth	V_0	0.54 [μm^3]	[2]
	Max cell growth rate	k_{max}	1.0 [h^{-1}]	[1]
	Cell biomass density	ρ	* [$kg_X\ m^{-3}$]	-
	Random noise in division volume	η_{div}	5.0 [%]	[2]
	Cell division orientation noise	η_{orient}	0.2 [%]	[2]
Toxin -Common	Per cell secretion rate	$k_{T,cell}$	* [$kg_T\ s^{-1}$]	-
	Lethal concentration	T_c	* ‡ [$kg_T m^{-3}$]	-
	Lysis delay	$1 / k_{lysis}$	0.125 [h]	[3]
	Weapon cost per unit secretion	c	0.0005 [h]	This study
Toxin - Contact	Extracellular needle length	$L_{needle} = R$	0.5μm	[3]
	Min. needle penetration for hit	$L_{penetration}$	0.01μm	[3]
	Number of hits to kill target cell	N_{hits}	1 [-]	Estimated from [4]
	Secretion rate	k_{sec}	0.0-500.0 [h^{-1}]	This study
Toxin - Diffusible	Toxin specific production rate	k_T	* [s]	-
	Toxin yield per unit biomass	α	* [$kg_T\ kg_X^{-1}$]	-
	Toxin diffusivity	D_T	$4x10^{-11}$ [m^2s^{-1}]	[5]
	Toxin Damköhler number	$\mathbb{D}_T = l^2 k_T \alpha \rho / D_T T_c$ $= k_{sec}\, l^3 / D_T\, N_{hits}$	0-3.5x10^{-3} [-]	This study

Domain	Diffusive boundary layer height	δ	25 [µm]	This study
	Domain width	L_x	300 c	This study
	Slougher height	$h_{slougher}$	40 [µm]	This study
Numerical	Mesh element size	h	5 [µm]	[2]
	Simulation timestep	Δt	0.025 [h]	[1]
	Cell / needle sorting grid size	h	10 [µm]	[3]
	Conjugate gradient absolute tolerance	e_{CG}	0.001 [-]	[1]
	Max. contact iterations	Max_{iter}	8 [-]	[1]
	Regularization weight	α	0.04 [-]	[2]
	Growth restriction factor	$1/\gamma$	0.002 [-]	[2]

Supplementary Table 3: Strains of *Pseudomonas aeruginosa* PAO1 constructed and used in this study.

Strain	Genotype	Purpose
WT	Wild-type	Attacker in CDI competitions
ΔCDI	Deletion of CDI1 (PA0040-PA0041)	Susceptible in CDI competitions
ΔR2	Deletion of pyocin R2 locus (PA0615-PA0628)	Remove pyocin R2 (tailocin)
ΔR2Δ*wbpL*	Deletion of pyocin R2 locus (PA0615-PA0628).<br>Deletion of *wbpL* (PA3145)	Susceptible in tailocin competitions.<br>CDI user in CDI vs tailocin competitions.
Δ*wapR*	Deletion of *wapR* (PA5000)	Attacker in tailocin competitions.
ΔR2Δ*wapR*	Deletion of pyocin R2 locus (PA0615-PA0628)<br>Deletion of *wapR* (PA5000)	Tailocin disarmed, assessment of fitness effect of wapR deletion
ΔCDIΔ*wapR*	Deletion of CDI1 (PA0040-PA0041)<br>Deletion of *wapR* (PA5000)	CDI susceptible tailocin user in CDI vs tailocin competitions.
ΔCDIΔR2Δ*wapR*	Deletion of CDI1 (PA0040-PA0041)<br>Deletion of pyocin R2 locus (PA0615-PA0628)<br>Deletion of *wapR* (PA5000)	Check that CDI still functions in Δ*wbpL* background.
ΔCDIΔR2Δ*wbpL*	Deletion of CDI1 (PA0040-PA0041)<br>Deletion of pyocin R2 locus (PA0615-PA0628)<br>Deletion of *wbpL* (PA3145)	Susceptible in double CDI + tailocin competitions.<br>Check that CDI still functions in Δ*wbpL* background.
ΔR2Δ*wbpL*+pSEVA524	Deletion of pyocin R2 locus (PA0615-PA0628).<br>Deletion of *wbpL* (PA3145)	Empty vector control for *wbpL* complementation.
ΔR2Δ*wbpL*+pSEVA524::wbpL	Deletion of pyocin R2 locus (PA0615-PA0628).<br>Deletion of *wbpL* (PA3145)	Complementation of *wbpL*.

Supplementary Table 4: Primers used for construction of deletion mutants and *wbpL* complementation.

Primer	Sequence
CDI1-out-F	agttcatgtccaatcaccacacc
CDI1-out-R	agggttcagttcgatgtaccgg
CDI1-del-UpF	aagcttctgcaggtcgactctagaggatccaagaagatcgaactggtcggc
CDI1-del-UpR	tgtacttcggcataatcagcctcctccagcgcgatggagagccacgatttc
CDI1-del-DownF	tcaaggaatgaaatcgtggctctccatcgctatatagttgagtaagctttgctgcga
CDI1-del-DownR	cccgtggaaattaattaaggtaccgaattcaagcgattccgatatcgtgtcg
R2-out-F	gaggctttccatggctgacc

R2-out-R	tgaggttgcaggtgacatcc
R2-del-UpF	aagcttctgcaggtcgactctagaggatccgatcacgccaacgaactggtc
R2-del-UpR	gatgggtttcaggcgtcacccccttgccgccagcctgttcaggcatggttg
R2-del-DownF	tcgaaggagtcaaccatgcctgaacaggctggcggcaaggggtgac
R2-del-DownR	cccgtggaaattaattaaggtaccgaattctacgttgtaccgaccgattccc
wapR-detect-F	ccatgtctatggcgccttcac
wapR-detect-R	aaccgcatccgtttcttcgtc
wapR-del-DownF	caagcttctgcaggtcgactctagaggatcgctactgggctacatcctgtac
WapR-del-DownR	taaggttgtagttatggcgctcgtcgatgagaagtactggaacgagaagaccttc
wapR-del-UpF	ccgcggaaggtcttctcgttccagtacttctcatcgacgagcgccataac
wapR-del-UpR	acccgtggaaattaattaaggtaccgaattcaagaatttcgaggcgatggc
wbpL-detect-F	ggctcagtatagccggtaagtc
wbpL-detect-R	caaatacgaggctgagcaggag
wbpL-del-DownF	caagcttctgcaggtcgactctagaggatccaggcggtgatagccaaagtg
wbpL-del-DownR	aaggttctctttccaatgatgatctggatgctcttggcggtaggatacaagg
wbpL-del-UpF	ggaacccgccttgtatcctaccgccaagagcatccagatcatcattggaaagagaac
wbpL-del-UpR	ACCCGTGGAAATTAATTAAGGTACCGAATTccgcctttgatctatgccaatg
wbpL-compF	tcggtacccgggctagatttaagaaggagatatacatatgatgatctggatgatcgcgtg
wbpL-compR	gtcgccagggttttcccagtcacgacgcggccgcattaggatttttccaaggaacccgc

Supplementary Table 5: Numbers of replicates and exact p-values

See separate file SupplementaryTable5.xlsx

Captions for supplementary videos

Movie S1: Comparison of short- and long-range weapons using agent-based modelling. Attackers armed with contact (blue) or diffusible (magenta) weapons compete against unarmed susceptible cells (yellow) within a 300µm-wide, 2-D biofilm. Representative 10h simulations are independently initialized with different attacker starting frequencies (rows from top to bottom: 1:9, 1:1, 9:1 attacker : susceptible ratio) and densities (left column: 10 cells; right column; 100 cells). Cells are removed once they reach the top edge (y=40µm) of the biofilm. Long-range (diffusing) toxins are only effective a high initial frequency (bottom row); by contrast, the short-range (contact) weapon is effective at all frequencies. Secretion rate k_{sec} equivalent to 100.0 h^{-1} for all scenarios shown. Where present, black contour indicates lethal concentration of diffusible toxin ($u_T = T_c$).

Movie S2: Comparison of short- and long-range weapons in invasion simulations. Low numbers of attacker cells, armed with contact (blue) or long-range (magenta) weapons, are randomly inserted into established 20µm-by-50µm 2-D biofilms containing unarmed susceptible cells (yellow). Rows depict different initial attacker frequencies (from top to bottom: 0.001, 0.01 and 0.1); columns depict different attacker secretion rates (from left to right: k_{sec} equivalent to 0.0, 20.0, 100.0 and 500.0 h^{-1}). Cells are removed once they reach the top edge (y=20µm) of the biofilm. Attacker lineages armed with contact weapons can establish at the base of the biofilm and then expand laterally by eliminating susceptible competitors; conversely, attackers armed with diffusible toxins are unable to increase their initial numbers to the point that the toxin becomes deadly, and are stochastically lost from the simulation. Simulations run for a maximum of 25h, terminating early if all attackers or all susceptible are lost.

Movie S3: Comparison of short- and long-range weapon simulations at different secretion rates. Attackers armed with contact (blue) or diffusible (magenta) weapons compete against unarmed susceptible cells (yellow) within a 300µm-wide, 2-D biofilm. Representative 10h simulations are independently initialized with different attacker secretion rates (rows from top to bottom: k_{sec} equivalent to 0.0, 20.0, 100.0 and 500.0 h^{-1}) and starting frequencies (columns from left to right: 1:9, 1:1, 9:1 attacker : susceptible ratio). Cells are removed once they reach the top edge (y=40µm) of the biofilm. Where present, black contour indicates lethal concentration of diffusible toxin ($u_T = T_c$).

Movie S4: Agent-based modelling of direct contests between the two weapons users. Here, mutually-susceptible attackers armed with contact (blue) and diffusible (magenta) weapons compete directly against each other. Simulated cells compete for space within a 300µm wide, 2D-biofilm, with cells removed from the top edge (y=40µm) of the biofilm. 10h simulations are initiated with a total of 10 (left column) or 100 (right column) cells, and with an initial ratio of contact-weapon-armed cells to diffusible-weapon-armed cells of 9:1 (top row), 1:1 (middle) or 1:9 (bottom). When starting at higher frequency, either attacker type can overcome the other, but at equal frequency it is the contact weapon user that wins. Where present, black contour indicates lethal concentration of diffusible toxin ($u_T = T_c$).

Extended Data Figures

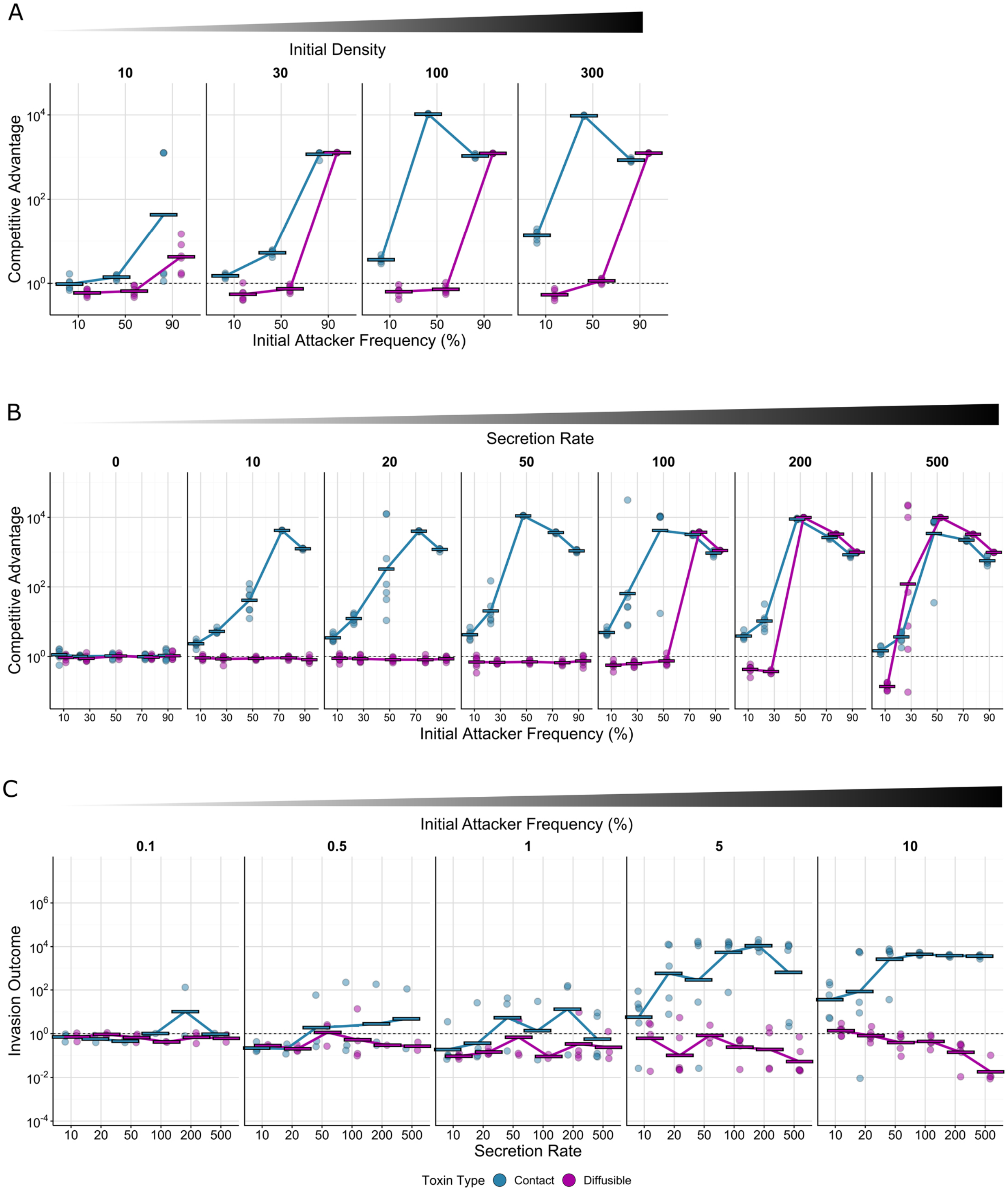

Extended data Figure 1: Agent-based modelling shows differences between weapons due to initial density of competitions, weapons depend differently on toxin secretion rate and that contact weapons better facilitate invasion than diffusible weapons at equivalent secretion rates. A Quantification of competition outcomes for all tested densities (secretion rate:

100). Densities of 10 and 100 cells correspond respectively to “Low” and “High” starting densities shown in Figure 1. Competitive advantage assesses the fold change in the attacker strain compared to its competitor from the beginning to end of the simulation (Methods). Horizontal lines indicate the mean from multiple simulations (n = 6). **B** Outcomes of competition simulations over a range of secretion rates and initial attacker frequencies (10%, 30%, 50%, 70%, 90%); initial density: 150 cells. Competitive advantage assesses the fold change in the attacker strain compared to its competitor from the beginning to end of the simulation (Methods). Horizontal lines indicate the mean from multiple simulations (n = 7). **C** Quantification of competition outcomes for invasions as a function of secretion rate. Invasion outcome is the same as competitive advantage (the fold change in the attacker strain compared to its competitor from the time of invasion to the end of the simulation). Horizontal lines indicate the mean from multiple simulations (n ≥ 6, see Table S5 for more details; only invasions where the invader was still present at the end of the simulation were analyzed).

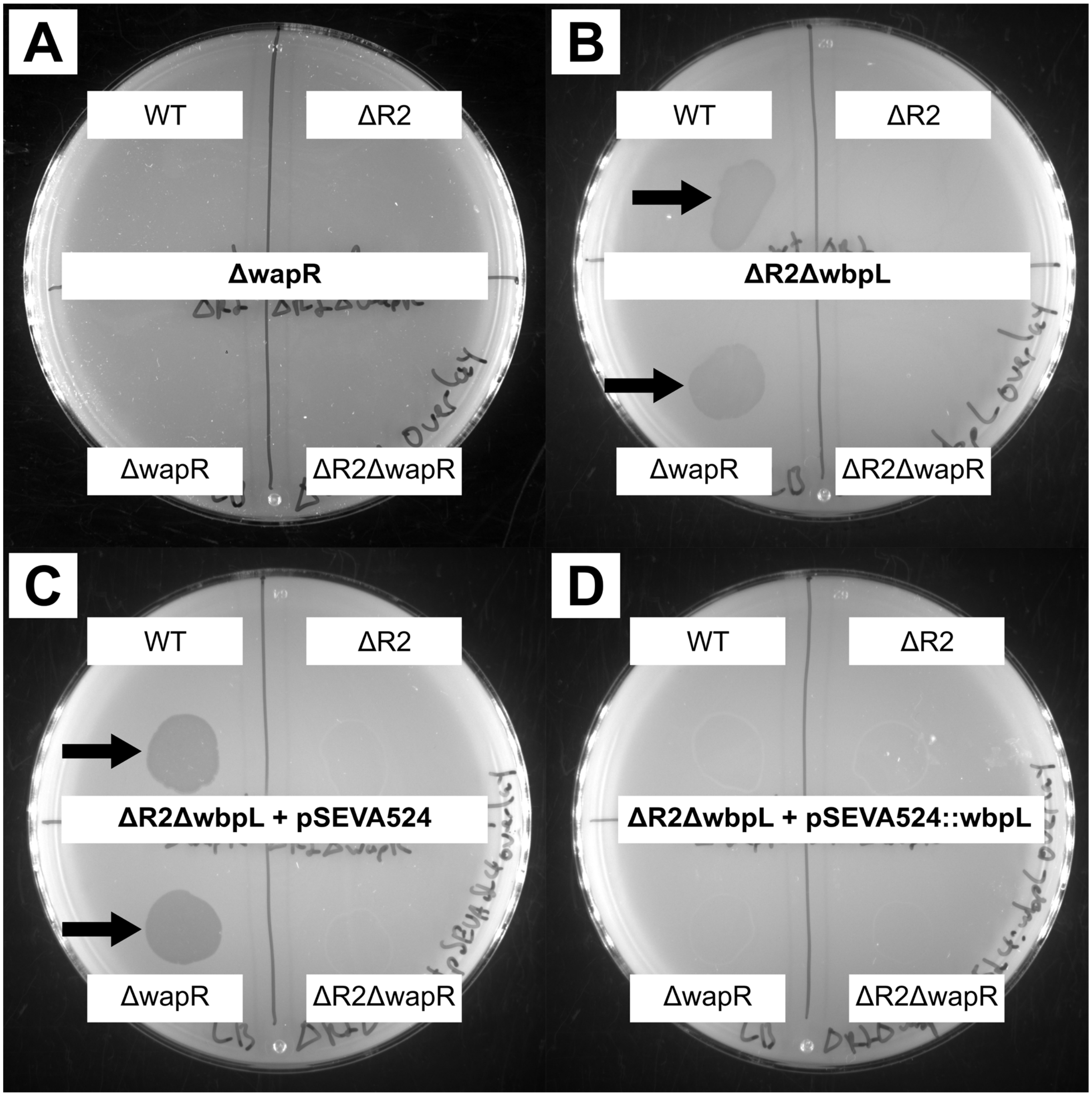

Extended data Figure 2: **Lipopolysaccharide biosynthesis genes affect susceptibility to pyocin R2.** Images of agar overlay assays showing pyocin R2 zones of clearing (arrows) for different LPS mutants. The strain in the overlay is indicated in the center of each plate. The source strain for the pyocin R2 is indicated at the corners. Pyocins were prepared by sterile filtering supernatant from overnight cultures. Overlays were prepared by mixing 1mL of overnight culture with 7mL 0.75% LB agar then thoroughly drying. **A** Δ*wapR* shows no zones of clearing. **B** ΔR2 Δ*wbpL* (the entire pyocin R2 gene cassette was first deleted from this strain, then *wbpL* deleted second) shows zones of clearing from WT and Δ*wapR*, but not when pyocin R2 is deleted. **C** Complementing ΔR2Δ*wbpL* with empty vector pSEVA-524 does not rescue clearing. **D** Complementing ΔR2Δ*wbpL* with pSEVA-524 carrying *wbpL* shows no clearing from WT or Δ*wapR*.

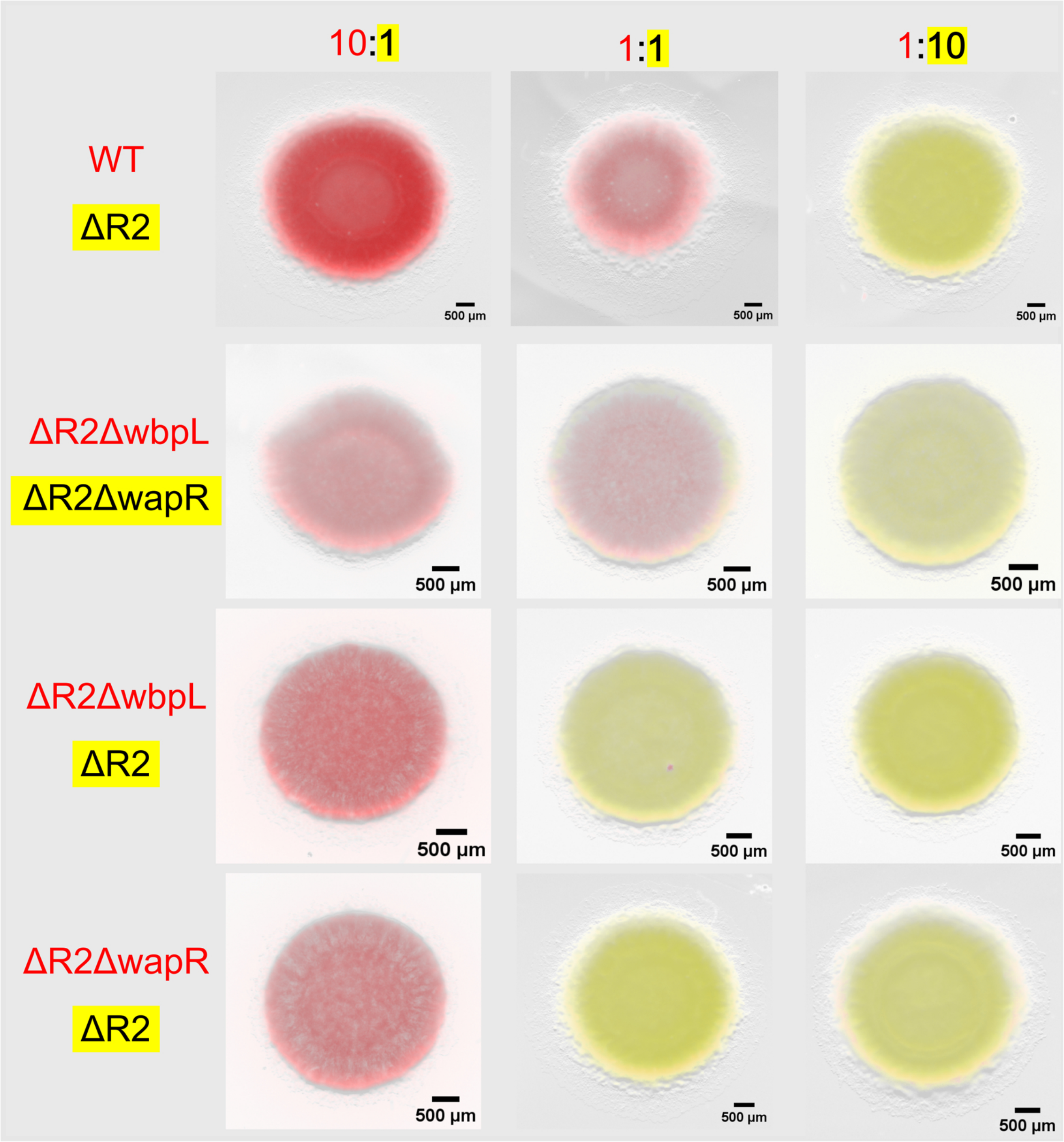

Extended data Figure 3: Lipopolysaccharide biosynthesis gene deletions affect competition outcomes in the absence of pyocin R2. Microscopy shows that the Δ*wbpL* strain cannot compete with wild-type *P. aeruginosa*, even in the absence of killing by tailocins (pyocin R2). Conversely, Δ*wbpL* and Δ*wapR* (second from top) are closely matched and look similar to wild-type competed against ΔR2 (top). Competitions were inoculated with ~2x10^6 cells/µL. Images are representative from three independent experiments.

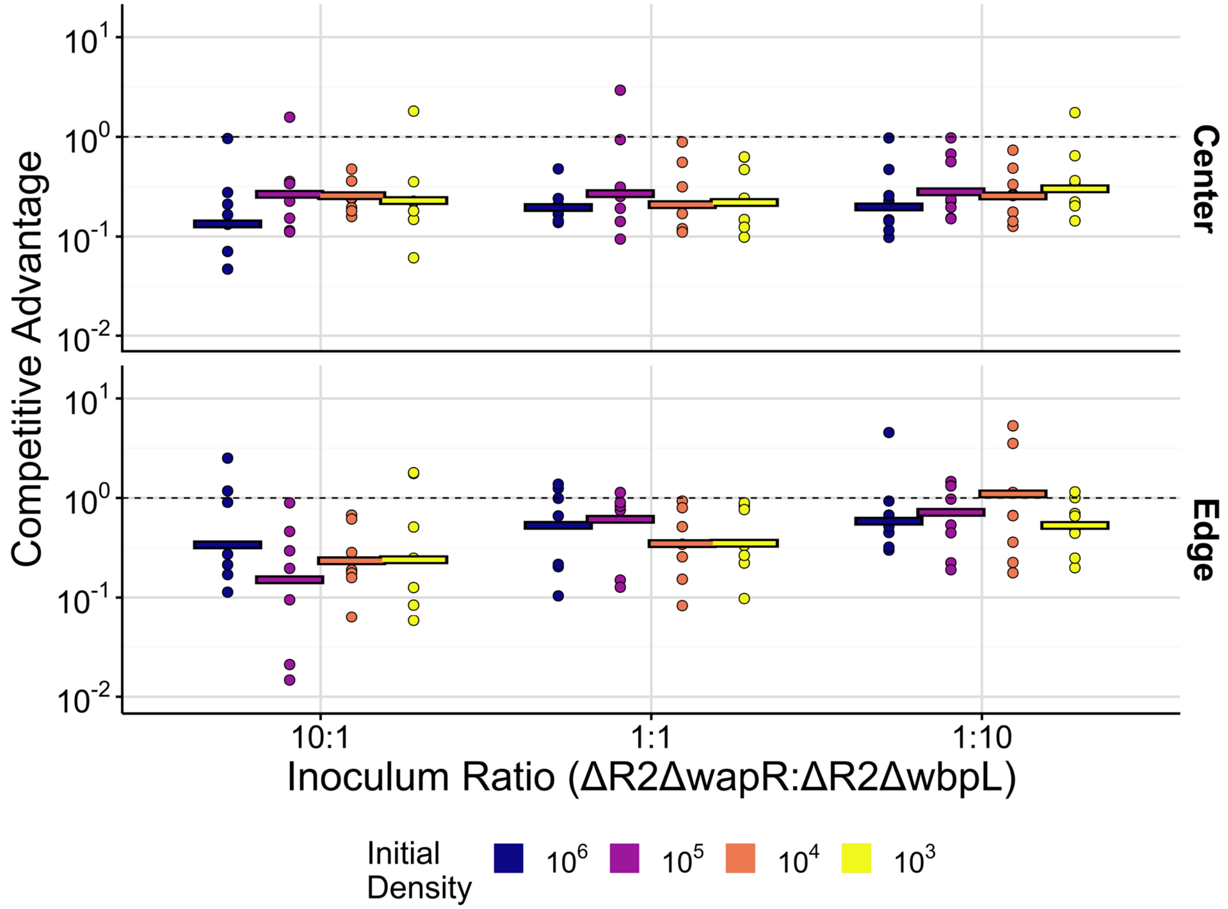

Extended data Figure 4: Deletion of lipopolysaccharide biosynthesis gene *wapR* causes a disadvantage compared to deletion of *wbpL* when both strains have pyocin R2 deleted. Quantification of colony competition outcomes between lipopolysaccharide biosynthesis mutants in the absence of pyocin R2. Colonies were inoculated at the stated initial ratios and densities (mean inoculum density 1.8 * 10^{3}, 10^{4}, 10^{5}, 10^{6} CFU/µL). Competitive advantage assesses the fold change in the attacker strain compared to its competitor from the beginning to end of the competition. Horizontal lines indicate the mean from biological replicates ($n \geq 6$, See Table S5 for exact n values). The mean (-0.637) across all replicates from all densities and inoculum ratios for the center was significantly different from 0 (One sided Welch's t-test, $t = 20.48$, $df = 111$, $p = 2.2e-16$), so was used as the baseline advantage of ΔwbpL over ΔwapR. This difference in advantage was subtracted from all competitions involving strains with these LPS biosynthesis gene deletions.

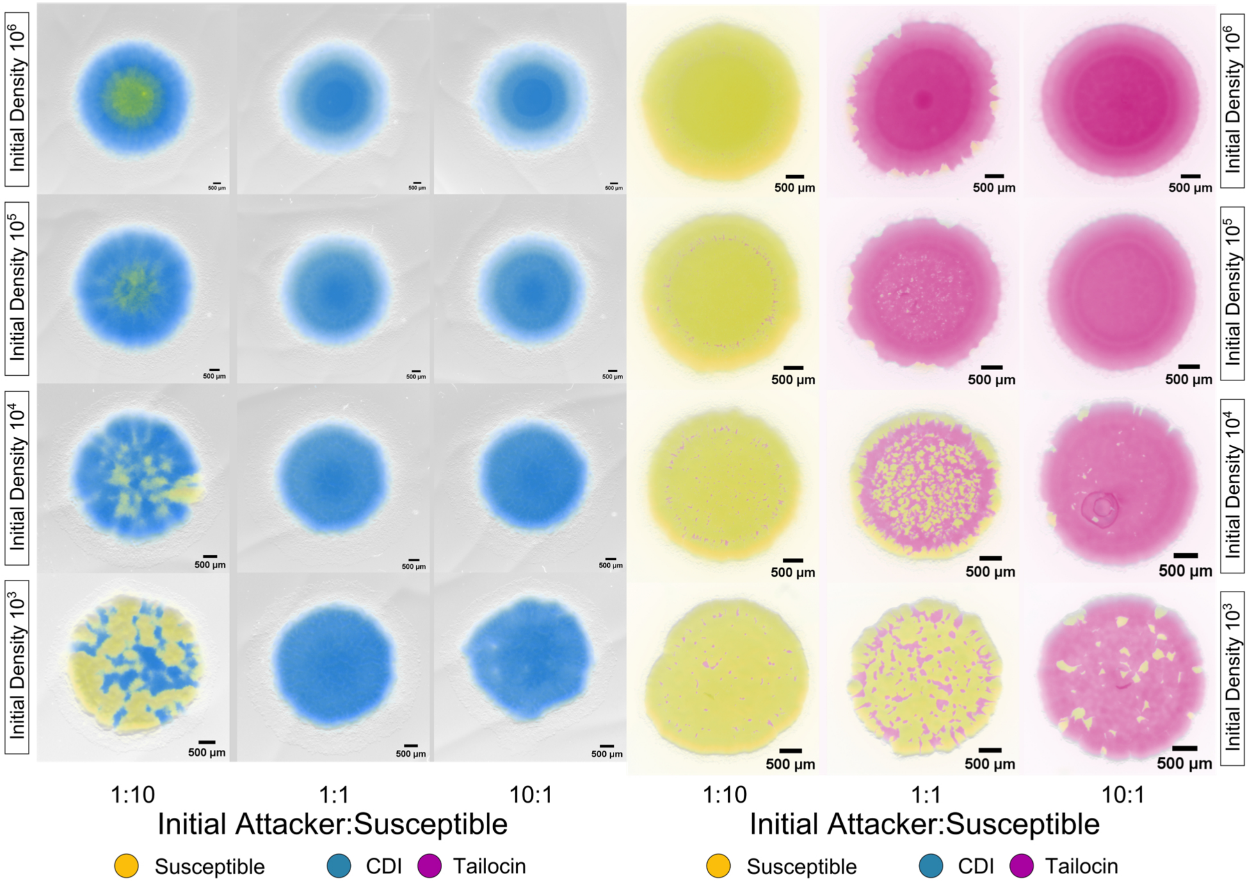

Extended data Figure 5: Colony competitions of contact dependent inhibition (CDI) and tailocins highlight differences between contact and diffusible toxins. Representative microscopy images of colony competitions inoculated from different starting densities (mean inoculum density 1.9 * 10^3, 10^4, 10^5, 10^6 CFU/µL). and initial ratios of attacker to susceptible cells taken after 48 h of growth. All strains are expressing constitutive fluorescent protein genes and false-coloured either blue (CDI attacker, top), magenta (tailocin attacker, bottom) or yellow (susceptible, top and bottom). Scale bar indicates 500 µm. For the CDI competitions the attacker was wild-type and the susceptible has the CDI toxin and anti-toxin deleted. For the tailocin competitions, the attacker is Δ*wapR* and the susceptible strain is ΔR2Δ*wbpL*. Images shown here are representative, images were taken for every colony sampled (data presented in Figure 2), exact values of n are detailed in supplemental table 5.

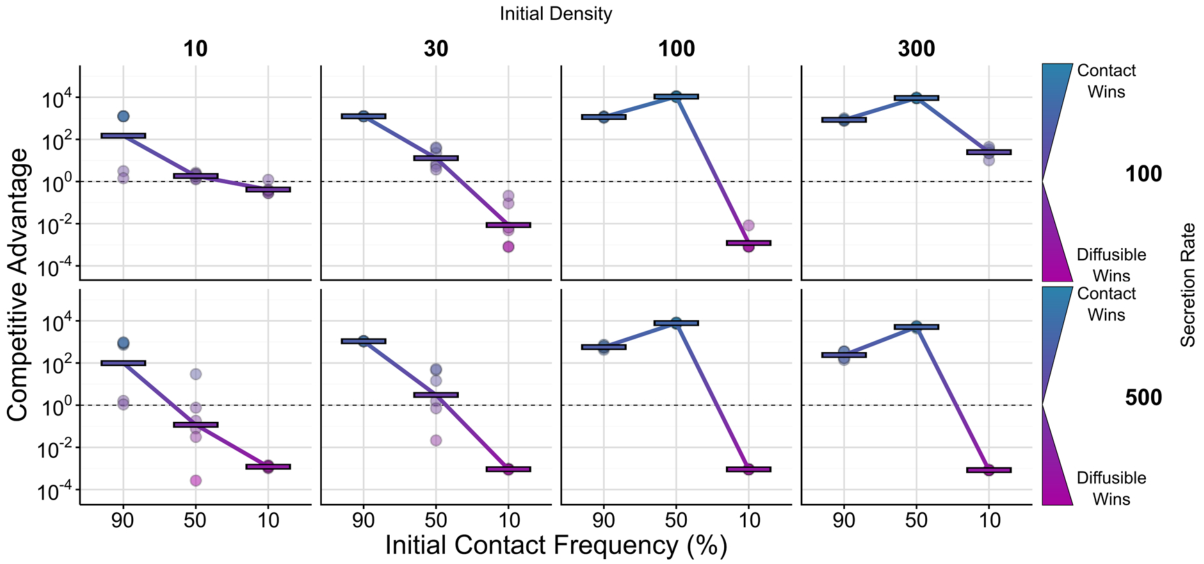

Extended data Figure 6: Agent-based modelling of head-to-head weapon competitions between short and long-range weapon users. Quantification of simulated direct weapon competition outcomes started at different initial densities, ratios and secretion rates. Density indicates the initial number of cells in the simulation. Competitive advantage assesses the fold change in the attacker strain compared to its competitor from the beginning to end of the competition. Horizontal lines indicate the mean from multiple simulations (n = 6). Densities of 10 and 100 cells, with secretion rate 100, correspond respectively to “Low” and “High” starting densities shown in Figure 3.

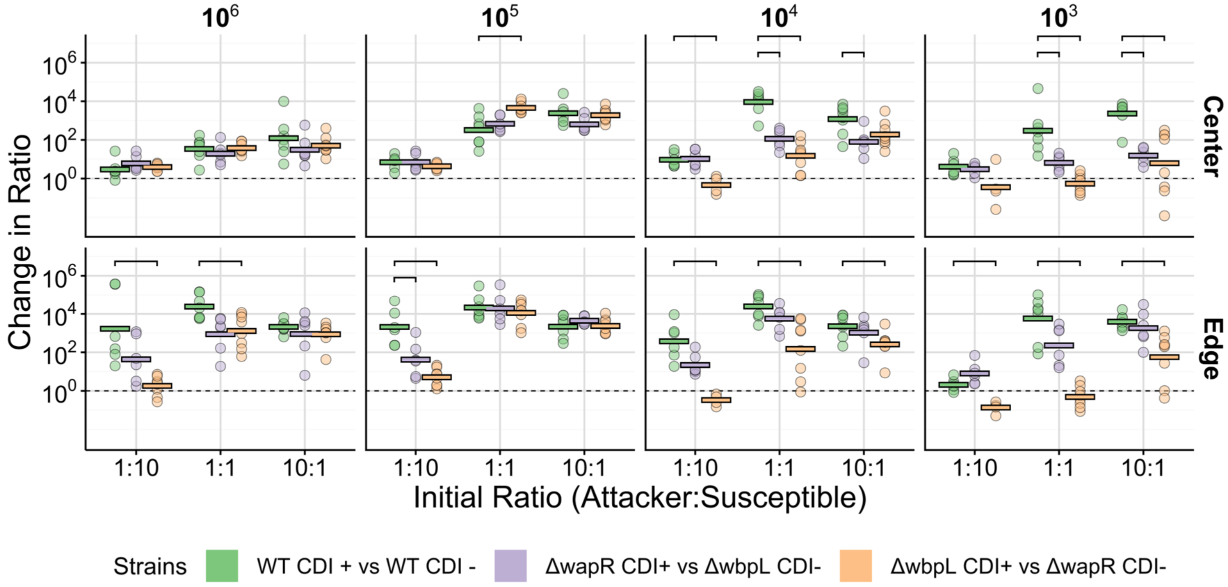

Extended data Figure 7: Outcomes of colony competitions shows that CDI remains functional in LPS biosynthesis gene mutants, but its effectiveness is diminished at low densities. Outcomes of CDI mediated competitions in wild-type (WT, green) or asymmetric LPS backgrounds. For these cases, both strains also have tailocins (pyocin R2) deleted. The attacking CDI+ strain is either Δ*wapR* against a CDI susceptible Δ*wbpL* (mauve) or CDI+ Δ*wbpL* against CDI susceptible Δ*wapR* (orange). Colonies were inoculated at the denoted initial densities (mean inoculum density 2.0 * 10^3, 10^4, 10^5, 10^6 CFU/µL) and quantified by sampling, plating and counting colony forming units after 48h of growth. Horizontal lines indicate the mean from biological replicates (n ≥ 4, see Table S5 for exact n values). Competitive advantage assesses the fold change in the attacker strain compared to its competitor from the beginning to end of the competition. Top brackets indicate a significant difference between each single weapon and the combination of weapons (two-sided Welch's t-test, $p < 0.05$, Benjamini-Hochberg correction for multiple testing, see Table S5 for exact p values).

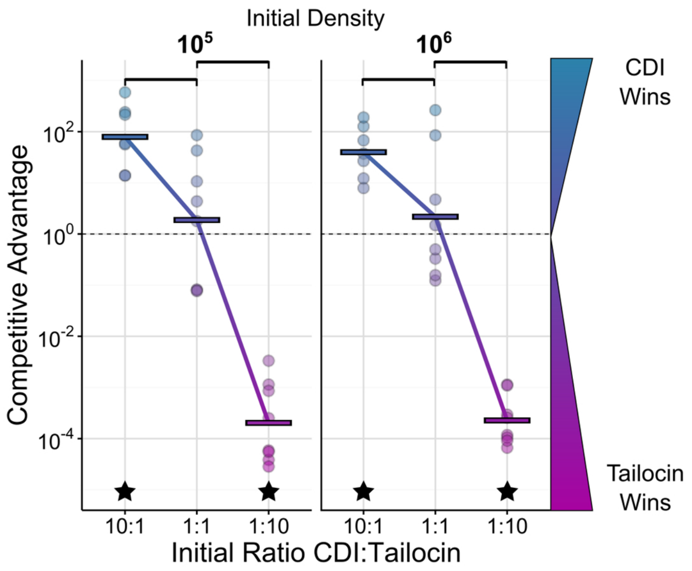

Extended data Figure 8: Head-to-head weapon competitions between short and long-range weapon users at the colony edge. Quantification of direct weapon colony competition outcomes at the colony edge by sampling, plating and counting colony forming units. Competitive advantage assesses the fold change in the attacker strain compared to its competitor from the beginning to end of the competition. Values above 1 (10^0, dashed line) indicate an advantage for CDI, while values below 1 (i.e. 10^{-2}, 10^{-4}) indicate an advantage for tailocins. Horizontal lines indicate the mean from biological replicates (n = 8). Top brackets indicate a significant difference between the initial ratios (two-sided Welch's t-test, $p < 0.05$, Benjamini-Hochberg correction for multiple testing, see Table S5 for exact p values). Stars indicate a competitive advantage significantly different from 0 (one-sided Welch's t-test, $p < 0.05$, Benjamini-Hochberg correction for multiple testing, see Table S5 for exact p values). Competitions were inoculated with different initial densities (mean inoculum density 2.3 * 10^5, 10^6 CFU/µL). The genotype of the CDI using, tailocin susceptible strain (blue) is ΔR2Δ*wbpL*. The genotype of the tailocin using, CDI susceptible strain is ΔwapRΔCDI.

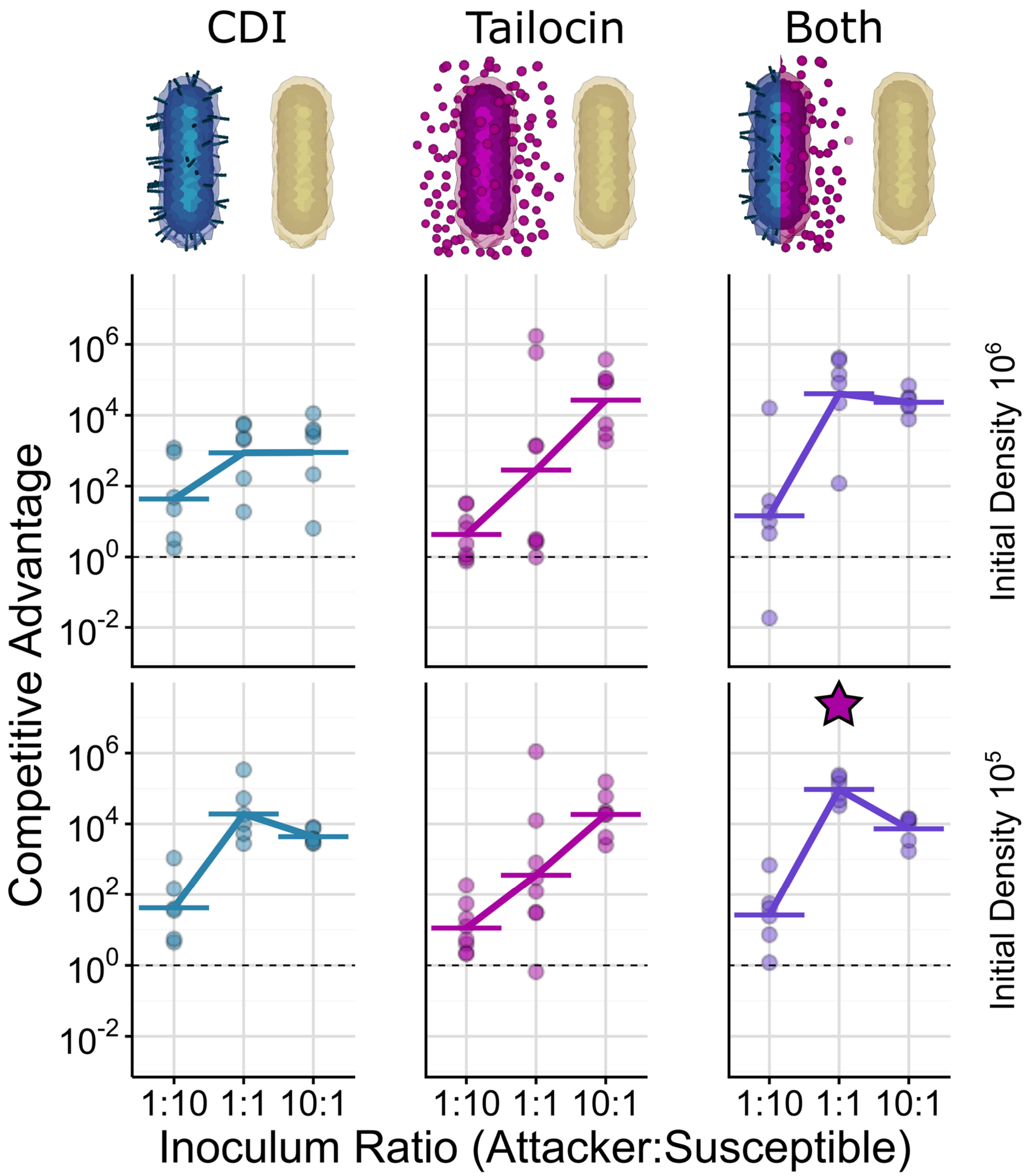

Extended data Figure 9: Short and long-range weapon benefits can combine positively at the colony edge. Quantification of competition outcomes in the colony edge for two initial cell densities (mean inoculum density 1.9 * 10^5, 10^6 CFU/μL). Competitive advantage assesses the fold change in the attacker strain compared to its competitor from the beginning to end of the competition. Competitions where the attacker has just CDI (blue, left), just tailocins (magenta, centre) or both weapons (purple, right) show the advantage gained from using two weapons together as compared to just one. Data are adjusted to account for differences in competitiveness of the strain backgrounds (Δ*wapR* relative to Δ*wbpL*; see methods and Extended data Figure 2).

Horizontal lines indicate the mean from biological replicates ($n \geq 6$, see Table S5 for exact n values).The star above the double weapon data indicates a significant difference between the combination of weapons and just tailocins (two-sided Welch's t-test, $p < 0.05$, Benjamini-Hochberg correction for multiple testing, see Table S5 for exact p values). Data from colony edge are noisier than in the colony center but patterns are consistent with the colony interior.

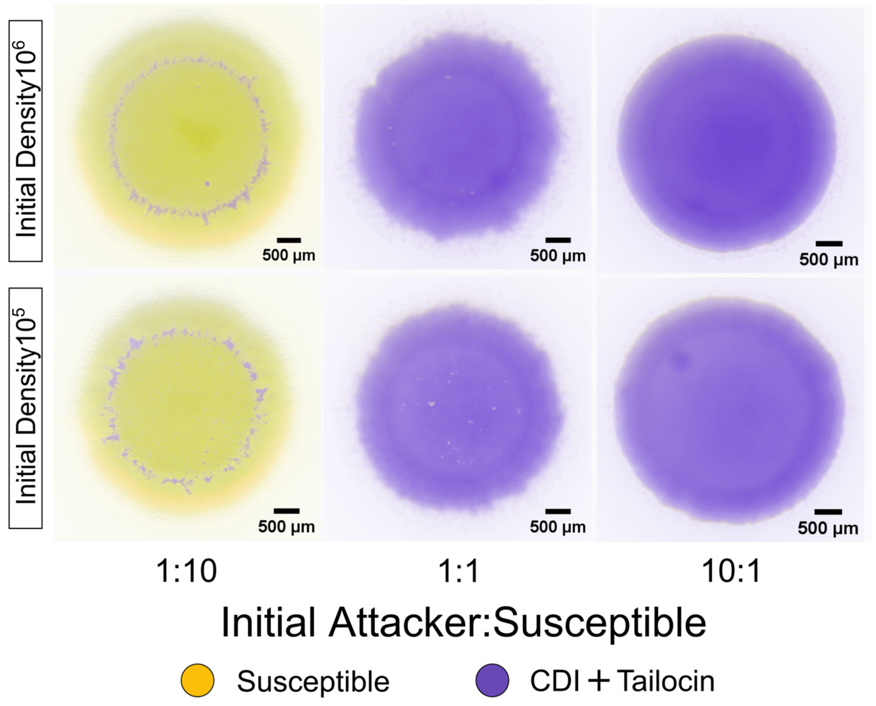

Extended data Figure 10: Microscopy images of colony competitions with doubly-armed attackers. Representative microscopy images (taken after 48 h of growth) of colony competitions inoculated from different starting densities (mean inoculum density 1.9 * 10^5, 10^6 CFU/µL). and initial ratios of attacking and dual CDI/tailocin susceptible cells. All strains are expressing constitutive fluorescent protein genes and false-coloured either purple (attacker) or yellow (CDI and tailocin susceptible). Scale bar indicates 500 µm. The genotype of the attacker is Δ*wapR*. The genotype of the susceptible strain is ΔCDIΔR2Δ*wbpL*. Images shown here are representative, images were taken for every colony sampled (data presented in Figure 4).

Supplementary References

1. Rudge, T. J., Steiner, P. J., Phillips, A. & Haseloff, J. Computational Modeling of Synthetic Microbial Biofilms. *ACS Synth. Biol.* **1**, 345–352 (2012).
2. Smith, W. P. J. *et al.* Cell morphology drives spatial patterning in microbial communities. *Proc. Natl. Acad. Sci. U.S.A.* **114**, (2017).
3. Smith, W. P. J. *et al.* The evolution of the type VI secretion system as a disintegration weapon. *PLOS Biology* **18**, e3000720 (2020).
4. Ringel, P. D., Hu, D. & Basler, M. The Role of Type VI Secretion System Effectors in Target Cell Lysis and Subsequent Horizontal Gene Transfer. *Cell Reports* **21**, 3927–3940 (2017).
5. Konisky, J. & Richards, F. M. Characterization of Colicin Ia and Colicin Ib: PURIFICATION AND SOME PHYSICAL PROPERTIES. *Journal of Biological Chemistry* **245**, 2972–2978 (1970).
